# Chronic sclerosing sialadenitis (Küttner's tumor) of the submandibular salivary gland: Our experience of one case report

**DOI:** 10.1002/ccr3.2303

**Published:** 2019-07-15

**Authors:** Anna Poghosyan, Martin Misakyan, Ani Sargsyan, Parandzem Khachatryan, Gayane Hakobyan

**Affiliations:** ^1^ Department of ENT and Maxillofacial Surgery “Heratsi” No. 1 University Hospital YSMU Yerevan Armenia; ^2^ Department of Pathological Anatomy and Clinical Morphology “Heratsi” No. 1 University Hospital YSMU Yerevan Armenia

**Keywords:** chronic sclerosing sialadenitis, IgG4, Küttner's tumor, submandibular salivary gland disease

## Abstract

Surgical excision of the submandibular salivary gland in patients with chronic sclerosing sialadenitis can be complicated because of hyalinosis of the surrounding soft tissues. Patients with autoimmune diseases suspected of having salivary gland neoplasms or granulomatous disease should be carefully investigated for chronic sclerosing sialadenitis to prevent potential harm.

## 1 INTRODUCTION

Chronic sclerosing sialadenitis (CSS) (Küttner's tumor) is a chronic long‐lasting inflammatory disease of the salivary glands, more often affecting submandibular salivary gland.^1^, ^2^, ^3^, ^4^ This relatively rare condition presents as a hard and enlarged mass that is clinically indistinguishable from a salivary gland neoplasm.^1^, ^2^ More recently, CSS has been regarded as an immunoglobulin G4 (IgG4)‐related idiopathic sclerosing lesion, which is frequently associated with regional lymph nodal adenopathy.^3^, ^4^

Most patients note recurrent pain, discharge, and swelling that is often associated with eating, but others only have an asymptomatic hard swelling of the submandibular gland.^1^, ^5^, ^6^ Clinically, CSS may present with a firm, painless neck mass, which mimics a neoplastic process or a painful enlarged gland, such as in sialadenitis.^1^, ^2^, ^3^, ^4^, ^5^, ^6^ Ultrasound features resemble those of a “cirrhotic liver” with diffuse involvement with multiple hypoechoic lesions against a heterogeneous background and duct dilatation.^7^, ^8^ At CT, the lesions usually display like homogeneous attenuation and enhancement, and on MR imaging, the lesions typically demonstrate homogeneous low‐to‐intermediate signal intensity.^9^ It may occur unilaterally with areas of calcification that may simulate malignancy. Lymphoma and the acute phase of Sjögren's syndrome should be included in the differential diagnosis.^9^

A case of submandibular gland CSS in a patient with autoimmune hemolytic anemia is presented.

## 2 CASE REPORT

A 67‐year‐old female patient was admitted to the clinic with a complaint of painful swelling of the right submandibular region. She had a 1‐year history of a hard, painless mass present in the submandibular region, which worsened in the 10 days prior to admission, with swelling of the submandibular region and fever. On palpation, the right submandibular salivary gland was hard and painful. Palpation and massage of the submandibular duct was painful, and there was no saliva evacuated from the duct.

**Figure 1:**
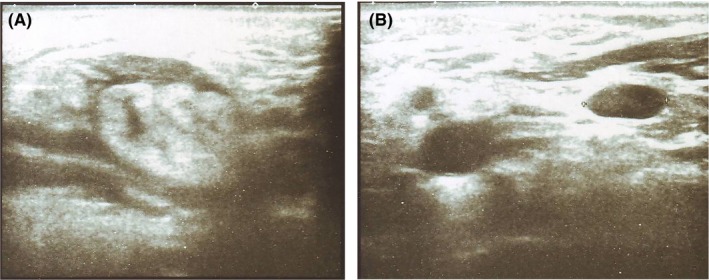
Ultrasound features of the (A) affected submandibular salivary gland and (B) lymph nodes

Ultrasound examination revealed enlargement of the right submandibular gland measuring 54.4 × 29.9 mm, with unclear borders. The echotexture revealed diffuse changes with low echogenicity, local anechoic areas, and hardened zones with severe peripheral vascularization. The gland was surrounded by a small amount of free fluid, and there was mild enlargement of the lymph nodes, measuring 13.0 × 13.3 mm (Figure 1A and B). Hepatomegaly and diffuse fatty liver were also found in this patient. She noted a 4‐year history of autoimmune hemolytic anemia and had been taking prednisolone prescribed by a hematologist for 3 years and ammonium glycyrrhizinate for the last year.

After a 7‐day course of antibiotic therapy, the patient noted reduction in pain and swelling. However, a new manifestation of inflammation was observed, and the patient was hospitalized with fever, pain, and swelling. The swelling of the submandibular space was firm, without clear boundaries. The color of the skin had not changed. Her hemoglobin (HGB) level was 109 × 10^9^ g/L, her red blood cell (RBC) count was −3.56 × 10^12^/L, her lymphocyte (LYMPH) percentage was 23.3%, and her erythrocyte sedimentation rate (ESR) was 50 mm/h. Submandibular gland extirpation by urgent indications was planned for this patient.

**Figure 2:**
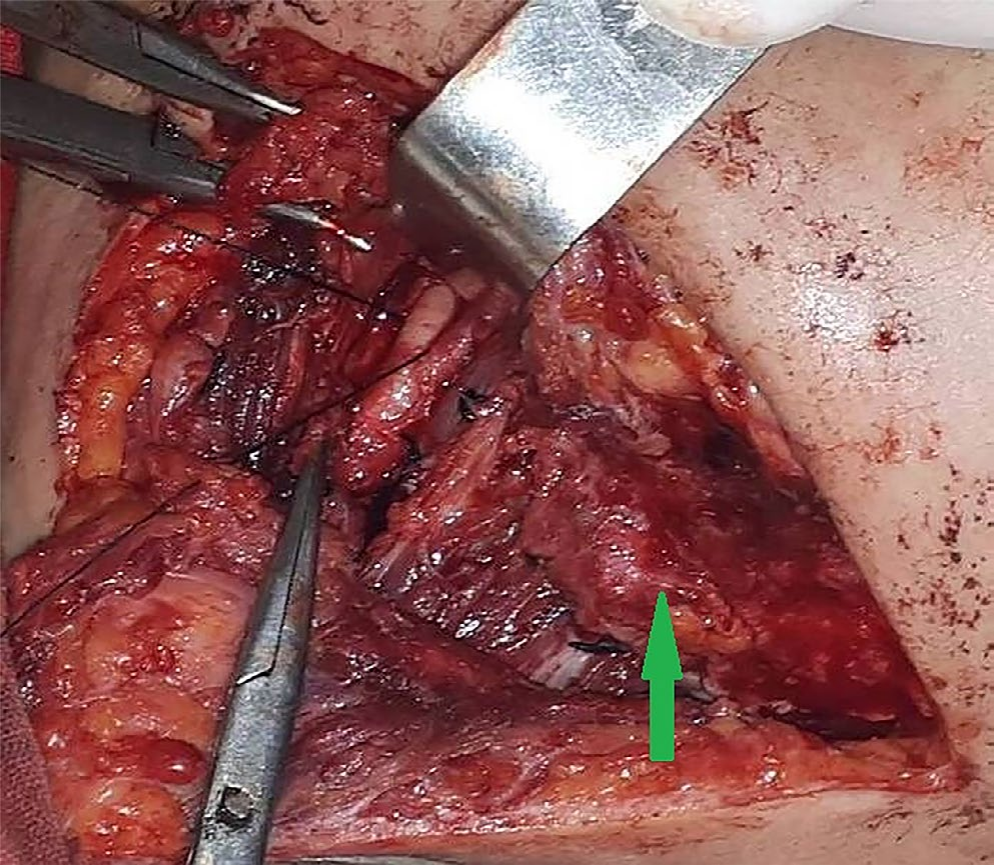
Cohesion with fibrosis and hyalinosis of the submandibular salivary gland with surrounding tissue and loss of anatomical boundaries: up arrow

**Figure 3:**
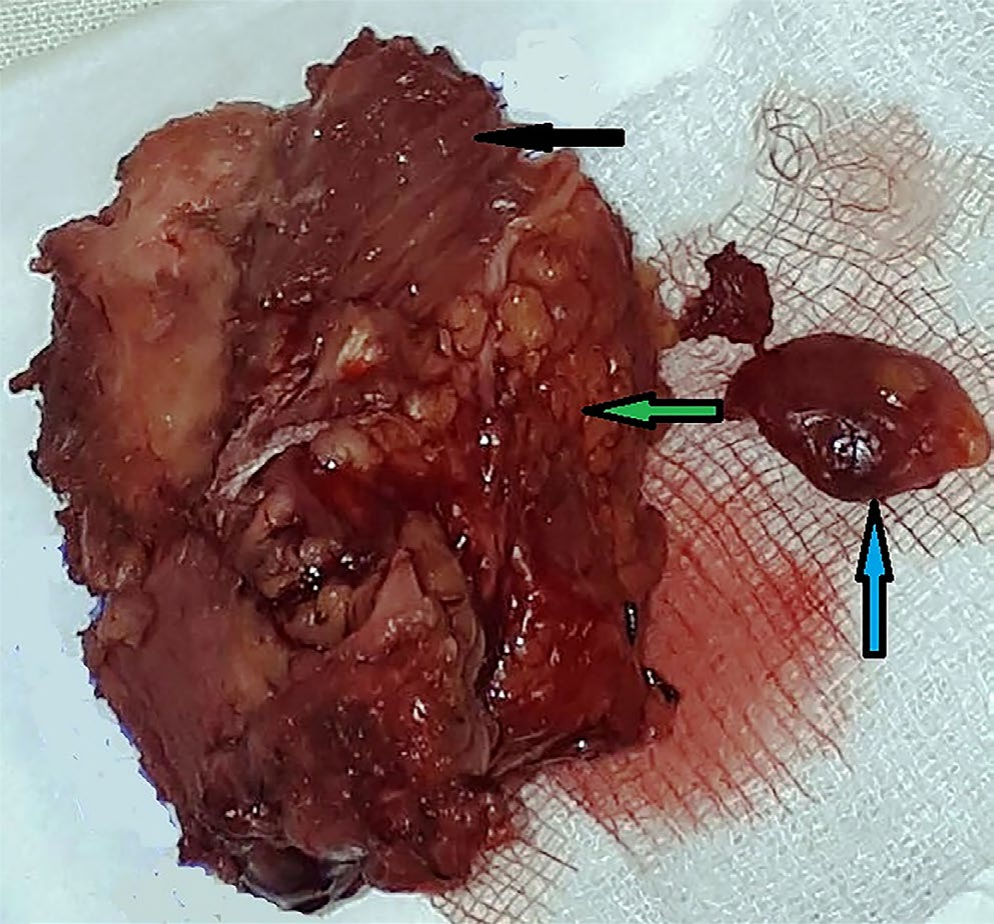
Extirpated submandibular gland in one block with the surrounding cohesive tissues, including muscles, fascia, and fatty tissue, the black arrow to the left shows welded muscles, the green arrow shows fatty tissue in conglomerate with the salivary gland, and the arrow pointing up shows an enlarged lymph node

**Figure 4:**
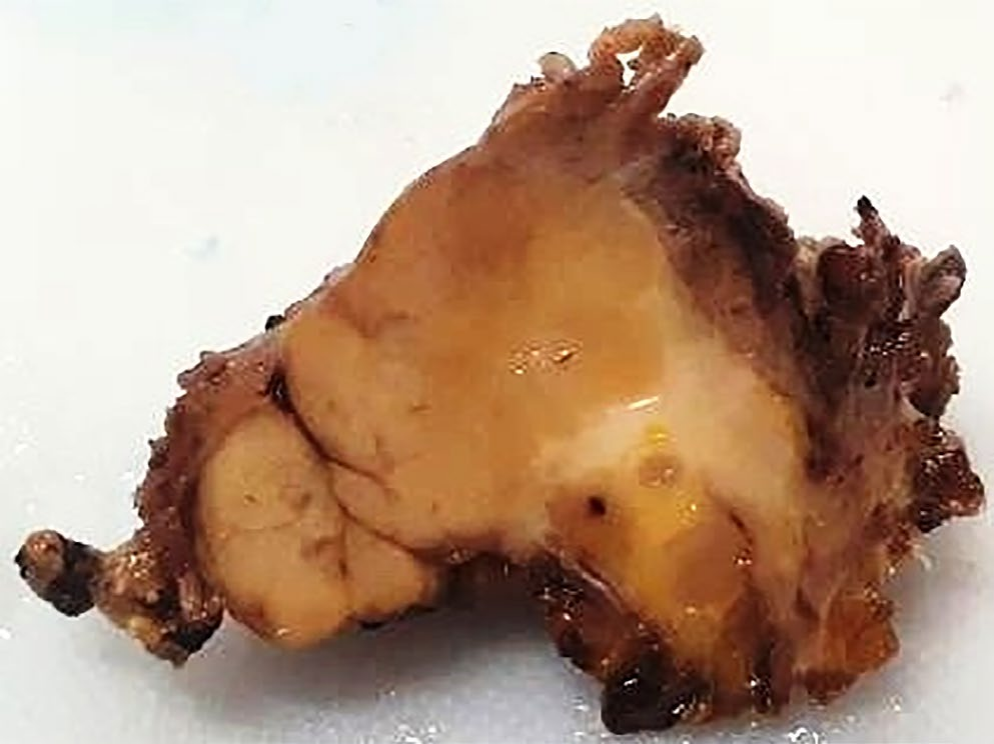
Cut surface of the submandibular salivary gland after formalin fixation showing a homogeneous consistency with a “pineapple‐like appearance due to the yellow and white inner layers and the hard, brownish rough coating”

Surgery was performed under general anesthesia. After incision of the skin and subcutaneous tissue, a hard mass with a rough surface without clear borders that was fused with the surrounding tissues (muscles, fascia, and blood vessels) was revealed (Figure 2). The adjacent muscles exhibited a hyaline‐like hardness and structure. There was no pus in the submandibular space. Clinically, the lesion appeared to be a malignant neoplasm. The classical horizontal incision for submandibular gland extirpation was modified by performing a vertical incision for better visualization of the affected area. Extirpation via resection of the submandibular gland conglomerate and enlarged lymph nodes was performed (Figure 3). Macroscopically, the gland measured 6.0 × 5.2 × 5.0 cm^3^. After formalin fixation, on the cut surface, the gland had a “pineapple‐like appearance with yellow and white layers” (Figure 4).

**Figure 5:**
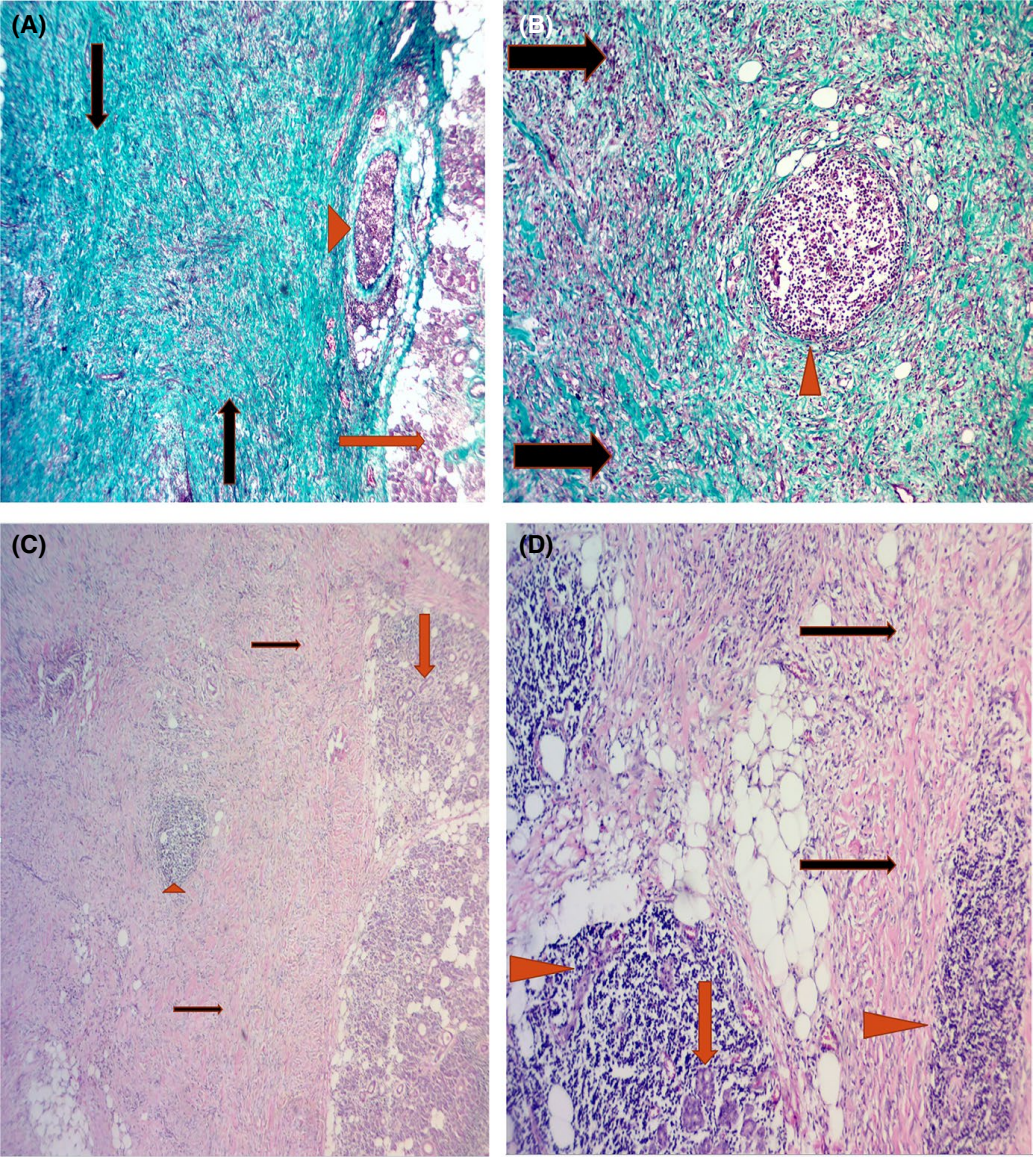
Histological view of the salivary gland showing (A) extensive periductal and perivascular fibrosis and sclerosis and foci of hyalinosis (Gomori trichrome original ×40 magnification): The red arrowheads show a lymphoid follicle; the red arrow shows atrophic acini; the black arrows show extensive fibrosis. B, Extensive fibrosis and lymphoid follicles (Gomori Trichrome ×100): The red arrowheads show the lymphoid follicle; the black arrows show extensive fibrosis. C, Extensive fibrosis in the salivary gland H&E ×40: The red arrowhead shows the lymphoid follicle; the red arrow show islands of the salivary gland; the black arrows show extensive fibrosis. D, Extensive fibrosis and lymphoid follicles with atrophic acini H&E ×100: The red arrowheads show the lymphoid follicle; the red arrow shows atrophic acini; the black arrows show extensive fibrosis

Microscopically, histological examination revealed extensive periductal and perivascular fibrosis and sclerosis, foci of hyalinosis, infiltration by mononuclear cells (mostly lymphocytes and plasma cells), acinar atrophy, and obliterative phlebitis (Figure 5A‐C). Thus, the histological diagnosis was CSS.

## 3 DISCUSSION

The exact etiology of CSS has not been established until now; however, various theories, such as salivary duct obstruction, salivary stasis, sialolithiasis, secretory dysfunction, and autoimmune response, have been proposed.^6^ In the present case, autoimmune hemolytic anemia was observed in the patient.

Although CSS is a chronic inflammatory condition, it presents clinically as a malignancy, for example, a painless hard mass in the submandibular gland, thus creating an interesting diagnostic dilemma.

The present patient had a long‐standing, painless, hard mass of the submandibular salivary gland that could manifest with features of submandibular salivary gland‐ and submandibular space‐specific infection, with symptoms of fever, pain, and firm subcutaneous tissues without skin hyperemia. The main problem in such cases is the possibility of an unclear preoperative diagnosis and unjustified scope of surgical intervention, which could occur in these patients. When the submandibular space is painful, diffuse and firm swelling in a patient with a high fever is not always responsible for space suppuration. In the present case, CSS exacerbation was manifested, which clinically mimicked acute specific infection, and the patient was operated on under urgent indications. However, there were no signs of acute inflammation intraoperatively.

There are a few cases in the literature that report regional lymph nodal adenopathy in the CSS of the submandibular gland. In the presented case, regional lymphadenopathy was also reported.

Ultrasound examination is a useful diagnostic tool and is initially used for the assessment of patients with enlarged salivary glands.^8^, ^9^ Ultrasonography features show diffuse heterogeneous echogenicity with multiple hypoechogenic shadows, similar to liver cirrhosis.^6^ These almost similar ultrasonographic features were described in the present case, where diffuse echostructure changed with low echogenicity, and hardened zones with severe peripheral vascularization were described. However, a review of the literature did not reveal that CSS can be diagnosed preoperatively with ultrasound. Chow et al reported cases of preoperative ultrasonography in six patients, with suspected CSS tumors in two patients.

On MR imaging, these lesions typically demonstrate homogeneous low‐to‐intermediate signal intensity on T2‐weighted images and low signal intensity on T1‐weighted images, with homogeneous enhancement. Areas of calcification that could be observed may simulate malignancy.^9^

FNA is frequently used to evaluate salivary gland neoplasms as it is a cost‐effective method with minimal risk to the patient.^10^ However, some cytopathological features of CSS may result in misinterpretation, as CSS lesions share some cytological features with inflammatory processes involving numerous lymphoid cells.^10^, ^11^ A portion of cytological specimens of CSS contain relatively large numbers of lymphoid cells and should be differentiated from malignant lymphoma arising from the submandibular gland.^11^ Leon et al report a series of four patients with CSS, and in each case, a discrepancy was found between the cytological diagnosis and the final histopathological diagnosis.

Surgically excised CSS lesions have a solid cut surface with preserved lobulations and fibrotic interlobular tissues that reflect the benign nature of the disease.^10^ Uncomplicated surgical removal of the salivary gland has been described in the literature.^1^, ^2^, ^5^, ^6^, ^10^, ^11^, ^12^, ^13^, ^14^, ^15^ In spite of this, in the present case, surgical excision of the gland was complicated because of severe fibrosis, hyalinosis, and cohesion of surrounding soft tissues, including muscles, fascia, and fatty tissue, with the submandibular gland, which results in a loss of gland boundaries. Thus, excision of gland and the involved surrounded tissue was performed in one block.

All cases described in the literature note that the diagnosis of CSS is mainly based on histopathological examination. Intraoperative frozen section examination may be a helpful tool for intraoperative diagnosis and prevention of unreasonably large dissections.^13^ The histological features of CSS show various characteristics, according to stage in the progressive process and the severity of inflammation. These features include interlobular cellular fibrosis, periductal inflammation, lobular chronic inflammation with numerous plasma cells, obliterative phlebitis, and florid follicular hyperplasia.^1^, ^2^, ^5^, ^6^, ^10^, ^11^, ^12^, ^13^, ^14^, ^15^, ^16^ The plasma cells are usually positive for IgG4 in most cases.^4^

According to Seifert, the four histological stages of CSS present corresponding to Stage 3: even more prominent lymphocytic infiltration, with lymphoid follicle formation, parenchymal atrophy, periductal hyalinization, and sclerosis. Squamous and goblet cell metaplasia may also be found in the ductal system.

Considering the fact that the present article describes one case report, the data may not be robust enough to explain complex issues, but this case could complement and refine the available data in the literature and provide additional information to explain this clinical situation.

## 4 CONCLUSION

Chronic sclerosing sialadenitis is a rare disease that is often clinically diagnosed as a malignant lesion. In the present case, the exacerbation period mimicked acute specific inflammation of the submandibular space, and urgent operative management was performed. Severe fibrosis and hyalinosis with the surrounded tissue made it possible to intraoperatively modify the operative technique and enlarge the volume of resection. CSS was diagnosed by the histopathologist. Patients with autoimmune diseases and salivary gland disorders that are suspicious for neoplastic or granulomatous disease should be carefully investigated for CSS to prevent potential harm.

## CONFLICT OF INTEREST

None declared.

## AUTHOR CONTRIBUTIONS

AYP: drafted the manuscript for important intellectual content and implemented the clinical work. MSM: made substantial contributions to the concept and design and was accountable for all aspects of the work in ensuring that questions related to the accuracy or integrity of any part of the work were appropriately investigated and resolved. AMS: was responsible for performing an accurate literature review and implementing the clinical work. PSK: made contributions to the design of the histological examinations. GAH: was responsible for the histological data, design, and literature review.

## ETHICAL CONSIDERATIONS

We confirm that explicit written consent to publish has been received from the described patient.
